# Situational Awareness of Influenza Activity Based on Multiple Streams of Surveillance Data Using Multivariate Dynamic Linear Model

**DOI:** 10.1371/journal.pone.0038346

**Published:** 2012-05-31

**Authors:** Eric H. Y. Lau, Calvin K. Y. Cheng, Dennis K. M. Ip, Benjamin J. Cowling

**Affiliations:** Department of Community Medicine and School of Public Health, Li Ka Shing Faculty of Medicine, The University of Hong Kong, Hong Kong, China; National Institutes of Health, United States of America

## Abstract

**Background:**

Multiple sources of influenza surveillance data are becoming more available; however integration of these data streams for situational awareness of influenza activity is less explored.

**Methods and Results:**

We applied multivariate time-series methods to sentinel outpatient and school absenteeism surveillance data in Hong Kong during 2004–2009. School absenteeism data and outpatient surveillance data experienced interruptions due to school holidays and changes in public health guidelines during the pandemic, including school closures and the establishment of special designated flu clinics, which in turn provided ‘drop-in’ fever counts surveillance data. A multivariate dynamic linear model was used to monitor influenza activity throughout epidemics based on all available data. The inferred level followed influenza activity closely at different times, while the inferred trend was less competent with low influenza activity. Correlations between inferred level and trend from the multivariate model and reference influenza activity, measured by the product of weekly laboratory influenza detection rates and weekly general practitioner influenza-like illness consultation rates, were calculated and compared with those from univariate models. Over the whole study period, there was a significantly higher correlation (ρ = 0.82, p≤0.02) for the inferred trend based on the multivariate model compared to other univariate models, while the inferred trend from the multivariate model performed as well as the best univariate model in the pre-pandemic and the pandemic period. The inferred trend and level from the multivariate model was able to match, if not outperform, the best univariate model albeit with missing data plus drop-in and drop-out of different surveillance data streams. An overall influenza index combining level and trend was constructed to demonstrate another potential use of the method.

**Conclusions:**

Our results demonstrate the potential use of multiple streams of influenza surveillance data to promote situational awareness about the level and trend of seasonal and pandemic influenza activity.

## Introduction

Many public health agencies routinely monitor influenza virus activity to facilitate situational awareness of the degree of disease activity in the community [1], [2], [3]. The importance of situational awareness has recently received more attention in biosurveillance [4]. Whereas one of the traditional uses of surveillance data is to identify peaks in disease incidence, the concept of situational awareness broadens this perspective so that surveillance data can be used to monitor disease trends in a range of situations. Much research has been done on the development of novel systems to complement traditional sources of surveillance data [5], [6] such as laboratory detections and sentinel influenza-like illness (ILI) surveillance in outpatients and inpatients. Recent examples include school absenteeism [7], online search counts [6] and over-the-counter medication sales [8]. Many studies have explored the choices of algorithms for sensitive, specific and timely detection of the start of a peak period of influenza activity in different settings [9], [10], [11], and shown that integration of multiple streams of surveillance data can improve performance [12], [13], [14]. Also, once entering the epidemic period, detection of the start of a peak becomes irrelevant and situation awareness of influenza activity will be more important for subsequent control measures. Few studies have explored the use of surveillance data to quantify levels and trends in disease activity through time thereby providing empirical support to situational awareness, particularly when multiple streams of data are available.

During the 2009 influenza pandemic, situational awareness was hindered by the introduction of new ‘drop-in’ surveillance systems and potential changes in behavior in pre-existing systems [15]. In this study we describe a multivariate statistical approach that permits the integration of multiple streams of influenza surveillance data to describe overall influenza activity in a single measure. We illustrate the performance of the model on past influenza seasons in Hong Kong, and show how a drop-in system during the 2009 pandemic could easily be incorporated to maintain good situational awareness of influenza activity.

## Methods

### Influenza surveillance data

The local Department of Health conducts influenza-like illness surveillance among a network of 50 private-sector sentinel general practitioners (GP) and 62 public-sector sentinel general outpatient clinics (GOPC) who report weekly proportion of outpatients fitting the surveillance definition of ILI (fever >38.5°C plus cough and/or sore throat [10], [12].) In February 2008 we established a school absenteeism monitoring system, with daily automated reporting of the proportion of students absent in 50 schools across the territory [16]. A limitation of school absenteeism data is the interruptions during regular school holidays, as well as school closures implemented to control influenza in 2008 and 2009 [17], [18]. The GOPC surveillance data were interrupted between mid-June 2009 and May 2010 when 8 designated flu clinics (DFCs) based within the GOPC sites were activated in place of regular GOPC services to manage the anticipated surge in patients during the pandemic. Sentinel outpatient clinics as well as other public and private outpatient clinics and hospitals routinely submit respiratory specimens from outpatients and inpatients to the Hong Kong Public Health Laboratory for surveillance and diagnostic purposes. Weekly data from the Public Health Laboratory on the proportion of submitted specimens with influenza virus detections reported by the Centre for Health Protection were available since January 1998. The product of the laboratory influenza detection rate and the GP ILI consultation rate was used as the reference standard indicator of influenza virus activity, rather than the laboratory data alone which suffer from denominator dilution during periods of non-influenza epidemics, and the GP ILI data alone which suffer from numerator dilution because not all ILI episodes are associated with influenza. Using the product of laboratory detection rates and ILI consultation rates can account for these issues and provide a more reliable measure of underlying influenza activity.

### Multivariate dynamic linear model

We fitted a multivariate dynamic linear time series model [19] to the three routine surveillance data streams, plus the drop-in DFC data available during the pandemic. Influenza isolation data was not available before 2004, but historical ILI data was available since 1998 and was used to parameterize the dynamic linear model. This is particularly important for obtaining reliable estimates in the initial period of 2004. For situational awareness, it is important to capture both the level and trend of the influenza activity to inform health planning and management. Hence we adopted a local linear specification which allows estimation of both aspects. We assumed that all data streams followed one underlying latent process with linear trend representing the (unobservable) true influenza activity. Thus each of the four streams contributed to estimation of the underlying level of influenza activity, and the underlying trend in activity. The influenza isolation rate was not included in the model as its availability is usually too late for the purpose of situational awareness. In this setting, the model was constructed under an unsupervised learning approach. The multivariate dynamic linear model is specified by the equations:
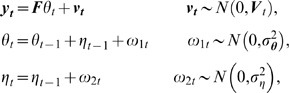
where ***y_t_*** are the observed surveillance data, ***F*** is the design matrix, *θ_t_* and *η_t_* are the level and trend of the latent process at time *t*, ***ν_t_*** is the observation error and *w_1t_* and *w_2t_* are the evolution error. Observation errors are assumed to be independent across data streams. Further details of the model are described in Text S1 and S2.

To demonstrate potential use of estimated level and trend, each were scaled to the range [0, 1] and averaged to create an overall influenza activity index reflecting influenza activity (details in Text S1). The index therefore takes higher values when activity is currently high or is increasing, and lower values when activity is low or decreasing. The index reflects two aspects of influenza activity in terms of level and trend which related to prevalence and transmissibility. However, optimization of the index is not pursued here. Missing data in the GOPC and school absenteeism data can be handled easily under the framework of the dynamic linear model [19]. All statistical analyses were performed in R version 2.12.0 (R Development Core Team, Vienna, Austria).

### Assessment of model performance

To assess the performance of the model in the context of situational awareness of influenza activity, we compared the estimated inferred influenza level and trend with the laboratory surveillance data in the same week, which is different from assessment of peak detection performance. More specifically, we assessed the correlation between inferred influenza level derived from the multivariate model and reference influenza activity as a measure of the ability to monitor influenza activity in real time. The correlations between inferred trends from the multivariate model and the trend in the reference influenza activity, estimated by the change between the subsequent and the preceding weeks, were also calculated. To compare the performance between the multivariate and univariate models, these measures were also calculated for each individual surveillance data stream and were statistically tested against those from the multivariate model. Distribution of the inferred influenza trend and level will also be plotted under different combinations of the level (low/medium/high) and trend (decreasing/stable/increasing) of the underlying influenza activity representing different phases of influenza epidemics.

## Results

**Figure 1:**
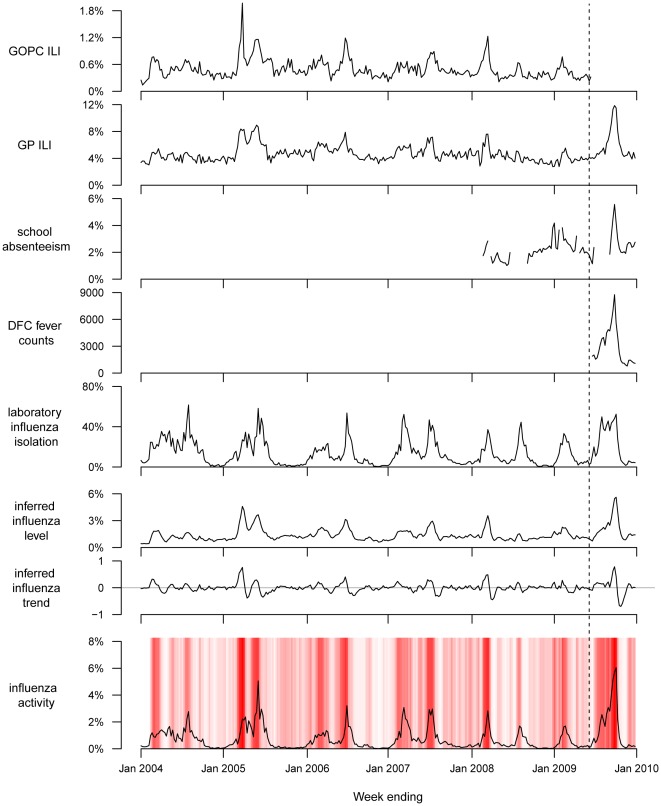
Surveillance data on influenza-like illness consultation rates in public General Outpatient Clinics (GOPC) and private general practitioners (GP), school absenteeism rates, and number of consultations with patients with febrile illness in Designated Flu Clinics which operated during the pandemic period; the inferred influenza level under the dynamic linear model based on the surveillance data streams and scaled to the range of the influenza activity proxy measure (product of laboratory influenza isolation rate and GP ILI rate); the inferred trend of influenza activity under the same model, scaled to the range [−1, 1]; the laboratory influenza detection rates from January 2004 through December 2009. The inferred influenza activity index was superimposed and color-coded from white (low) to red (high) in each panel. The vertical dashed line indicates the start of the pandemic period.

For each week since 2004, we estimated the latent level and trend, and calculated the overall influenza activity index based on all available GOPC, GP ILI, school absenteeism and DFC fever counts surveillance based on data available up to that week. The data along with the scaled inferred influenza level and trend from the dynamic linear model, and the reference influenza activity are shown in Figure 1. Superimposed strips represent the overall influenza activity index, with darker colors representing higher values of the index suggesting greater activity at that point or in the short-term future. The median of the index was 0.21 (range: 0.01–0.94) in the whole study period and 0.60 (range: 0.17–0.94) when influenza activity is greater than 2%. In general the index reflected activity and was able to capture most of the peaks in the laboratory data, while generating some false signals (e.g. 2005 autumn).

**Figure 2:**
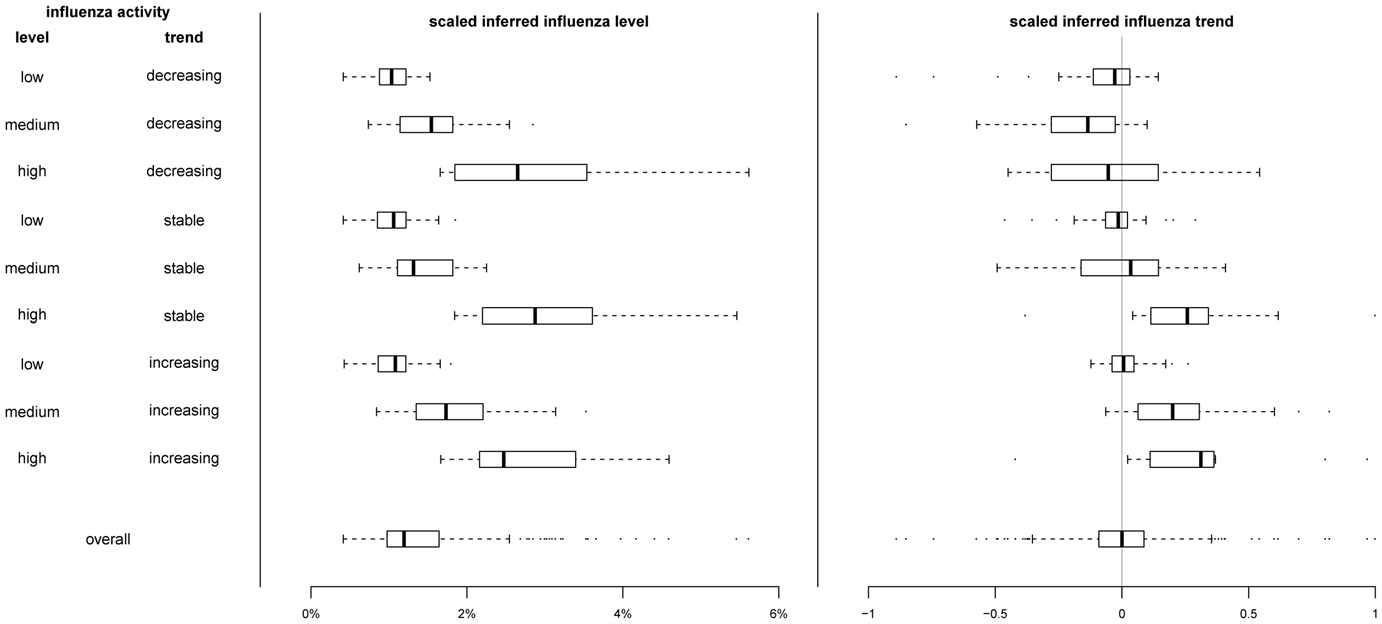
Box-plot of inferred influenza level and trend based on multivariate dynamic linear model utilizing four surveillance data streams including influenza-like illness consultation rates in public General Outpatient Clinics (GOPC) and private general practitioners (GP), school absenteeism rates, and number of consultations with patients with febrile illness in Designated Flu Clinics, under different patterns of influenza activity. Influenza activity was defined as low, medium or high if it is lower than 0.5%, between 0.5–2%, or higher than 2% respectively, defined as decreasing, stable or increasing if the percentage change between the following and preceding week is lower than −30%, between −30–30% or higher than 30% respectively. The inferred influenza level was scaled to the range of the influenza activity proxy measure (product of laboratory influenza isolation rate and GP ILI rate), while the inferred trend of influenza activity under the same model was scaled to the range [−1, 1].

**Figure 3:**
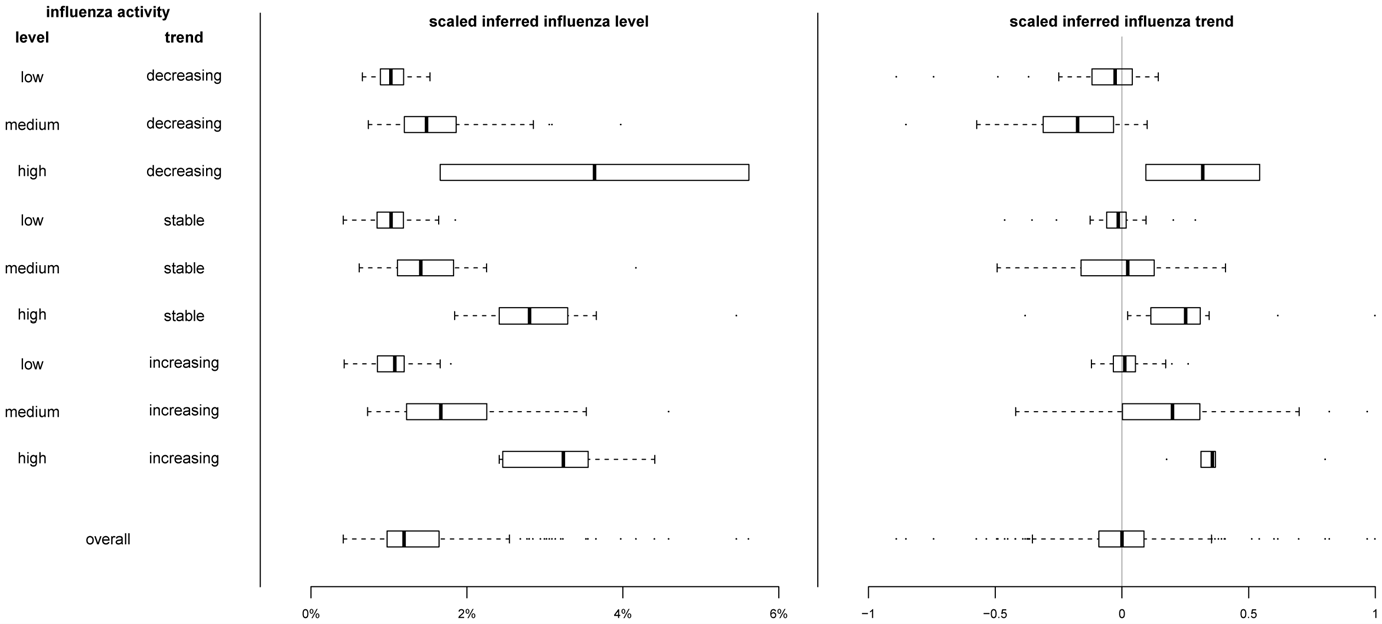
Box-plot of inferred influenza level and trend based on multivariate dynamic linear model utilizing four surveillance data streams including influenza-like illness consultation rates in public General Outpatient Clinics (GOPC) and private general practitioners (GP), school absenteeism rates, and number of consultations with patients with febrile illness in Designated Flu Clinics, under different patterns of influenza activity. Influenza activity was defined as low, medium or high if it is lower than 0.4%, between 0.4–2.5%, or higher than 2.5% respectively, defined as decreasing, stable or increasing if the percentage change between the following and preceding week is lower than −40%, between −40–40% or higher than 40% respectively. The inferred influenza level was scaled to the range of the influenza activity proxy measure (product of laboratory influenza isolation rate and GP ILI rate), while the inferred trend of influenza activity under the same model was scaled to the range [−1, 1].

We fitted dynamic linear models to the individual surveillance data streams, and estimated the correlation of these individual models versus influenza activity. We compared this with the correlation between the multivariate models using all available data versus influenza activity (Table 1). While the multivariate model captured the general pattern of influenza activity (Figure 1), it could not replicate the sharp peak in the influenza activity as shown by the moderate correlations (Table 1). However it significantly outperformed univariate models in capturing the underlying level of influenza activity over the study period (Table S1). The inferred influenza trend from the multivariate models correlated moderately with the change in influenza activity for the whole period (Table 2). It also reflected the change in influenza activity as good as the best of the other surveillance data streams in each period (Table 2 & Table S2). Figure 2 and 3 show the distributions of the inferred influenza level and trend under different patterns of influenza activity. While the inferred influenza trend partially captured the underlying trend, especially when influenza activity is low, the inferred influenza level followed the underlying influenza activity level closely.

**Table 1:** Correlations of GOPC, GP ILI rate, school absenteeism, DFC fever counts and inferred influenza level from the dynamic linear model with the influenza activity* in pre-pandemic, pandemic and the whole period, January 2004–December 2009.

	correlation† with influenza activity		
surveillance data	pre-pandemic period (Jan 2004–May 2009)	pandemic period (mid-Jun–Dec 2009)	whole period (Jan 2004–Dec 2009)
1. GOPC ILI‡	0.70	-	0.70
2. GP ILI	0.67	0.93	0.77
3. School absenteeism§	0.32	0.67	0.61
4. DFC fever counts¶	-	0.51	-
Inferred influenza level from model based on 1+2+3	0.75	0.93	0.82
Inferred influenza level from 1+2+3+4	-	0.94	0.82

DFC designated fever clinic; GOPC general outpatient clinic; GP general practitioner; ILI influenza-like-illness.

* Influenza activity measured by GP ILI consultation rate×laboratory influenza isolation rate.

† Correlations between surveillance data and laboratory isolation rate were calculated by fitting a univariate dynamic linear model to each data stream, and an overall multivariate model to all data streams.

‡ GOPC data were interrupted during the pandemic period due to the opening of designated flu clinics.

§ School absenteeism data were occasionally interrupted by school holidays or school closures. Correlations were calculated excluding data during the summer holidays.

¶ 8 designated fever clinics were activated in place of GOPCs to treat outpatients with influenza-like illness from mid-June 2009 to May 2010.

**Table 2:** Comparison of inferred trend from individual surveillance data and from model based on GOPC ILI rate, GP ILI rate and school absenteeism rate with changes in influenza activity*.

	correlation† with % change in influenza activity between the subsequent and preceding week		
surveillance data	pre-pandemic period (Jan 2004–May 2009)	pandemic period (mid-Jun–Dec 2009)	whole period (Jan 2004–Dec 2009)
1. GOPC ILI‡	0.42	-	0.42
2. GP ILI	0.30	0.11	0.24
3. School absenteeism§	0.45	−0.13	0.24
Inferred influenza trend from model based on 1+2+3	0.42	0.28	0.38
Inferred influenza trend from 1+2+3+DFC fever counts	-	0.29	0.38

DFC, designated fever clinic; GOPC, general outpatient clinic; GP, general practitioner; ILI, influenza-like-illness.

* Influenza activity measured by GP ILI consultation rate×laboratory influenza isolation rate.

† Correlations between surveillance data and the log ratios were calculated by fitting a univariate dynamic linear model to each data stream, and an overall multivariate model to all data streams. DFC was excluded from the analysis due to insufficient data for estimation of the inferred trend.

‡ GOPC data were interrupted during the pandemic period due to the opening of designated flu clinics.

§ School absenteeism data were occasionally interrupted by school holidays or school closures. Data during the summer holidays were excluded.

## Discussion

We have demonstrated the use of a multivariate method to integrate information from multiple streams of influenza surveillance data to improve situational awareness of the current level of influenza activity, and how a combined use of both the inferred influenza level and trend or an integrated index that can be potentially used to indicate overall influenza activity currently. Our study showed the advantage of a multivariate model-based approach especially if some surveillance data streams are interrupted or supplemented by additional systems during certain critical periods such as the 2009 influenza pandemic. During the whole study period, the estimated influenza level from the multivariate model showed higher correlation with influenza activity and the estimated influenza trend stably reflected the change in influenza activity as good as the best individual surveillance data streams from which it was derived (Table 1 & 2). In both the pre-pandemic and pandemic period, the inferred trend and level from the multivariate model was able to match, if not outperform, the best individual surveillance data albeit with missing plus drop-in and drop-out of different surveillance data streams. The multivariate time series approach can flexibly incorporate data from drop-in surveillance systems to improve performance and maintain situational awareness. Moreover, the dynamic linear model can handle missing data in a straightforward manner while allowing for serial autocorrelation and short-term or longer-term trends (details in Text S1). If particular surveillance systems were thought to provide information with higher quality, the relative importance of individual data streams could also be adjusted in the dynamic linear model by re-weighting different data streams.

A potential caveat of the method is that historical data are needed to parameterize the model prior to estimation of the overall influenza activity. The statistical time series model may not be able to fully describe the evolution of an infectious disease such as influenza. In this study we did not try to optimize the parameters for combining the level and trend into the overall influenza index nor validate the index with respect to an objective function. The level or trend may have different importance in various contexts which can be easily adjusted according to specific purpose, and compared to the simple average there may be superior combinations of these two parameters to provide a single prospective estimate of the degree of influenza activity currently and in the short-term future. We used the product of laboratory influenza isolation rate and GP ILI rate as a proxy measure for the reference influenza activity, which may partly explain the high correlation between GP ILI and influenza activity during the pandemic with particularly high proportion of medical consultations due to influenza. While we have taken into account the elevated ILI consultations during the pandemic which may have affected the laboratory isolation rate, change in healthcare consultation behavior was not accounted for due to limited data.

## Supporting Information

Text S1 Description of the multivariate dynamic linear model.
(DOC)

Text S2 Example syntax for estimation of the latent level and trend by the multivariate dynamic linear model.
(DOC)

Table S1 Test of differences in correlations with influenza activity, between surveillance data and inferred influenza level from the multivariate models.
(DOC)

Table S2 Test of differences in correlations with changes in influenza activity, between surveillance data and inferred influenza level from the multivariate models.
(DOC)
