# Supplementary material for: Situational Awareness of Influenza Activity Based on Multiple Streams of Surveillance Data Using Multivariate Dynamic Linear Model
Source: PLoS One. 2012 May 31;7(5):e38346. doi: 10.1371/journal.pone.0038346 (PMC3364986; doi:10.1371/journal.pone.0038346)
Supplement: Text S1 — Description of the multivariate dynamic linear model. (DOC) [file pone.0038346.s001.doc]

**Supporting Information**

**Description of the multivariate dynamic linear model**

The multivariate dynamic linear model [19] is a form of state space model for time series data with multiple observations. Dynamic linear model was chosen to accommodate the intrinsic serial correlation of infectious disease data, the observed mildly irregular seasonal pattern which usually violated the stationarity assumption, as well as missing data due to interruption of surveillance. In these models, observed data ***y_t_*** (representing a vector of data at time *t*) are assumed to be related to some underlying latent sequential process, or system. Allowing for this underlying system where subsequent (unobserved) system values *θ_t_* and *η_t_* are highly correlated, the consecutive observations ***y_t_*** are assumed to be conditionally independent. The design matrix ***F*** links each of the observed data to the same underlying latent process. With a Bayesian approach under this structure, multivariate normal distribution theory allows simple parameter estimation for some of the parameters. Formally, the model with local linear trend of the latent process is described by the following equations:

***y_t_*** *=* ***F*** *θ_t_ +* ***ν_t_*** where *ν_t_ ~ N(0,* ***V_t_****)*,

*θ_t_ = θ_t-1_ + η_t-1_ + w_1t_* where *w_1t_ ~ N(0, σ_θ_^2^)*, and

*η_t_ = η_t-1_ + w_2t_* where *w_2t_ ~ N(0, σ_η_^2^)*, and

*θ_t_* and *η_t_* are the level and trend of the latent process at time *t*, where in our study it is the underlying influenza activity. The observation error ***ν_t_*** and the ‘evolution’ errors *w_1t_* and *w_2t_* are internally and mutually independent, and the latent process and the observation variance matrix ***V_t_*** may be estimated from the data using a Kalman filter i.e. without requiring maximization of a likelihood function. The evolution variance *σ_θ_^2^* and the design matrix ***F*** were estimated by maximum likelihood method before applying the Kalman filter. *σ_η_^2^* contols the smoothness of the latent trend and was pre-specified to be 5000 in our study. Noninformative priors with Normal distributions were used at *t* = 0 which were then updated recursively by the Kalman filter. Data are assumed to be missing completely at random for school holidays and missing at random due to school closure or pandemic, which can be accommodated by practically setting the corresponding component in ***F*** to 0 so that the Kalman filter is updated based on the previous predictive distribution only. Operationally the filtering distribution of *θ_t_* is replaced by the one-step-ahead predictive distribution at the previous time point. To mimick the practical situation, for estimation of influenza activity at week *t*, only data available up to week *t* will be used to fit the dynamic linear model.

In our specific application where we model four surveillance data streams, i.e. GOPC, GP ILI rate, school absenteeism and DFC fever counts data, we assumed a working uncorrelated structure for the observation variance matrix, which was shown to be adequate in a similar context (11). The uncertainty in each individual surveillance data was incorporated into the model by specifying the relative variances in the following form:

***V_t_*** = *σ*^2^ $\left(\begin{matrix} v_{1t} & 0 & 0 & 0 \\ 0 & v_{2t} & 0 & 0 \\ 0 & 0 & v_{3t} & 0 \\ 0 & 0 & 0 & v_{4t} \end{matrix}\right)$.

We fixed ***V_t_*** ≡ ***V*** to be time invariant, but dynamic linear model allows time varying ***V_t_*** which is particularly useful when there are changes in sample size or quality in individual data stream. *ν_1t_*, *ν_2t_* and *ν_3t_* were specified relative to the mean variances in each surveillance data assuming binomial errors and *ν_4t_* assuming poisson errors for the fever counts. In our study, we set (*ν_1t_*, *ν_2t_*, *ν_3t_*, *ν_4t_*) = (1, 30, 7, 8 × 10^13^).

Having fitted the model for data at times 1,…, *t*, we extracted *θ_t_* and *η_t_* as an estimator of the level and trend of the underlying influenza activity. The overall influenza index at week *t* was generated iteratively by combining the latent level and trend with the following formula, based on data up to week *t*. As a demonstration of the use of combining level and trend into an overall index, we arbitrarily transformed each of the inferred level and trend to the range [0, 1] and used their average as the overall index. More specifically, we set index_t_ *=* [*p_Lt_* + logit(*p_Tt_*)^T^] / 2, where *p_Lt_* and *p_Tt_* are the percentile rank of the empirical distribution of *θ_t_* and *η_t_* respectively based on the estimates up to week *t*. logit(*p_Tt_*)^T^ are the logit transformation of the percentile ranks *p_Tt_* linearly transformed to the range [0, 1]. The overall index ranges from 0 to 1 and take values close to 1 when both the overall influenza activity is high and increases rapidly. The index was not optimized with respective to an objective function but used as a simple transformation and integration of level and trend into an index in the range of [0, 1].
