# Supplementary material for: Situational Awareness of Influenza Activity Based on Multiple Streams of Surveillance Data Using Multivariate Dynamic Linear Model
Source: PLoS One. 2012 May 31;7(5):e38346. doi: 10.1371/journal.pone.0038346 (PMC3364986; doi:10.1371/journal.pone.0038346)
Supplement: Text S2 — Example syntax for estimation of the latent level and trend by the multivariate dynamic linear model. (DOC) [file pone.0038346.s002.doc]

**Example syntax for estimation of the latent level and trend by the multivariate dynamic linear model**

require(dlm) ## use package ‘dlm’ in R

## surveillance data saved in table ‘surveillance.data’

n.streams <- 4

evol.var <- c(1, 30, 7, 8e13) ## relative variances of the evolution errors

sigma.eta <- 5000

n.burnin <- 52 ## number of weeks for burn-in estimation period of the DLM

n.week <- nrow(surveillance.data)

## Specification of the multivariate DLM local linear model

dlm.multi <- function(par) {

dlm(FF=matrix(c(exp(par[1:n.streams]), rep(0,n.streams)), nrow=n.streams, byrow=F), V=diag(evol.var)*exp(par[n.streams+1]),

GG=matrix(c(1,1,0,1), nrow=2, byrow=T), W=diag(c(exp(par[n.streams+2]), sigma.eta)), m0=rep(0, 2), C0=diag(rep(1e7, 2)))

**}**

## Function to fit the DLM model

dlm.fit <- function(surveillance.data, n){

dlm.mle.par <- dlmMLE(surveillance.data[1:n,-1], rep(-5, n.streams+2), dlm.multi)$par

dlm.model <- dlm.multi(dlm.mle.par)

dlm.filter <- dlmFilter(surveillance.data[,-1], dlm.model)

c (dlm.filter$m[n+1,1], dlm.filter$m[n+1,2])

}

## Save latent level and trend iteratively in the matrix ‘output.dlm’

output.dlm <- matrix(NA, ncol=2, nrow=n.week)

colnames(output.dlm) <- c("latent.level", "latent.trend")

for (i in (n.burnin+1):n.week){

output.dlm[i, ] <- dlm.fit(surveillance.data, i)

}

Sample dataset ‘surveillance.data’:

| week_ending | gopc | gp | school | dfc |
| --- | --- | --- | --- | --- |
| … |  |  |  |  |
| 5/23/2009 | 0.0073 | 0.0866 | 0.0157 | NA |
| 5/30/2009 | 0.0082 | 0.0922 | 0.0162 | NA |
| 6/6/2009 | 0.0096 | 0.0940 | 0.0151 | NA |
| 6/13/2009 | 0.0087 | 0.0897 | 0.0184 | NA |
| 6/20/2009 | NA | 0.1021 | 0.0211 | 1085 |

…
