# Supplementary material for: Situational Awareness of Influenza Activity Based on Multiple Streams of Surveillance Data Using Multivariate Dynamic Linear Model
Source: PLoS One. 2012 May 31;7(5):e38346. doi: 10.1371/journal.pone.0038346 (PMC3364986; doi:10.1371/journal.pone.0038346)
Supplement: Table S2 — Test of differences in correlations with changes in influenza activity, between surveillance data and inferred influenza level from the multivariate models. (DOC) [file pone.0038346.s004.doc]

**Table S2.** Test of differences in correlations with changes in influenza activity*, between surveillance data and inferred influenza level from the multivariate models.

| p-values, test of differences† in correlations‡ | | | | | |
| --- | --- | --- | --- | --- | --- |
|  | Pre-pandemic period  (Jan 2004 – May 2009) |  | Pandemic period  (mid-Jun – Dec 2009) |  | Whole period  (Jan 2004 – Dec 2009) |
|  |  | | | | |
| Surveillance data | Compare with multivariate model based on 1+2+3 | | | | |
| 1. GOPC ILI | 1.00 |  | - |  | 1.00 |
| 2. GP ILI§ | <0.01 |  | 0.14 |  | <0.01 |
| 3. School absenteeism¶ | 0.40 |  | <0.01 |  | 0.34 |
|  |  |  |  |  |  |
| Surveillance data | Compare with multivariate model based on 1+2+3+DFC fever counts | | | | |
| 1. GOPC ILI | 1.00 |  | - |  | 1.00 |
| 2. GP ILI§ | <0.01 |  | 0.14 |  | <0.01 |
| 3. School absenteeism¶ | 0.40 |  | <0.01 |  | 0.33 |

DFC designated fever clinic; GOPC general outpatient clinic; GP general practitioner; ILI influenza-like-illness.

*Influenza activity measured by GP ILI consultation rate х laboratory influenza isolation rate.

†Dependence between inferred trend from surveillance data and inferred influenza trend from the multivariate model was adjusted. For each test, data was excluded for the period with missing surveillance data.

­‡Correlations between trends in surveillance data and change in laboratory isolation rate were calculated by fitting a univariate dynamic linear model to each data stream.

­§GOPC data were interrupted during the pandemic period due to the opening of designated flu clinics.

¶School absenteeism data were occasionally interrupted by school holidays or school closures. Correlations were calculated excluding data during the summer holidays.
